# Clinical Features Associated with Strongyloidiasis in Migrants and the Potential Impact of Immunosuppression: A Case Control Study

**DOI:** 10.3390/pathogens9060507

**Published:** 2020-06-23

**Authors:** Angela Martinez-Pérez, Manuel Jesús Soriano-Pérez, Fernando Salvador, Joan Gomez-Junyent, Judith Villar-Garcia, Miguel Santin, Carme Muñoz, Ana González-Cordón, Joaquín Salas-Coronas, Elena Sulleiro, Dolors Somoza, Begoña Treviño, Rosángela Pecorelli, Jaume Llaberia-Marcual, Ana Belén Lozano-Serrano, Llorenç Quinto, Jose Muñoz, Ana Requena-Méndez, on behalf of the STRONG-SEMTSI working group

**Affiliations:** 1 CAP Casanova, Consorci d’Atenció Primaria en Salut Barcelona Esquerra, 08036 Barcelona, Spain; angelam@clinic.cat; 2 Barcelona Institute for Global Health, ISGlobal-Hospital Clinic, Universitat de Barcelona, 08036 Barcelona, Spain; llorenc.quinto@isglobal.org (L.Q.); jose.munoz@isglobal.org (J.M.); 3 Tropical Medicine Unit, Hospital de Poniente, El Ejido, 04700 Almería, Spain; manueljesus.soriano@ephpo.es (M.J.S.-P.); joaquin.salas@ephpo.es (J.S.-C.); anabelen.lozano@ephpo.es (A.B.L.-S.); 4 Department of Infectious Diseases, Vall d’Hebron University Hospital, PROSICS Barcelona, 08035 Barcelona, Spain; fmsalvador@vhebron.net; 5 Department of Infectious Diseases, Bellvitge University Hospital-IDIBELL; University of Barcelona, L’Hospitalet de Llobregat, 08907 Barcelona, Spain; jgomezj@bellvitgehospital.cat (J.G.-J.); msantin@bellvitgehospital.cat (M.S.); 6 Infectious Diseases Department, Hospital del Mar-IMIM, 08003 Barcelona, Spain; JVillar@parcdesalutmar.cat; 7 Department of Microbiology, Hospital Sant Pau, 08001 Barcelona, Spain; cmunoz@santpau.cat (C.M.); JLlaberia@santpau.cat (J.L.-M.); 8 Department of Infectious Diseases, Hospital Clinic, 08036 Barcelona, Spain; AGONZAL1@clinic.cat; 9 Department of Microbiology, Vall d’Hebron University Hospital, PROSICS Barcelona, 08035 Barcelona, Spain; esulleiro@vhebron.net; 10 Department of Microbiology, Hospital Universitari Bellvitge, 08907 Barcelona, Spain; dsomoza@bellvitgehospital.cat; 11 Tropical Medicine Unit Vall d’Hebron-Drassanes, PROSICS Barcelona, 08035 Barcelona, Spain; btrevino.bcn.ics@gencat.cat; 12 Internal Medicine Department, Hospital Universitario General de Catalunya, 08915 Barcelona, Spain; rpecorelli.capozzi@gmail.com; 13 Centro de Investigação em Saúde de Manhiça, 1929 Maputo, Mozambique; 14 Division of Infectious Diseases, Department of Medicine-Solna, Karolinska Institutet, 17177 Solna, Sweden

**Keywords:** *Strongyloides stercoralis*, migration, risk factors, case-control, immunosuppression

## Abstract

*Strongyloides stercoralis* is a widely distributed nematode more frequent in tropical areas and particularly severe in immunosuppressed patients. The aim of this study was to determine factors associated with strongyloidiasis in migrants living in a non-endemic area and to assess the response to treatment and follow-up in those diagnosed with the infection. We performed a multicenter case-control study with 158 cases and 294 controls matched 1:2 by a department service. Participants were recruited simultaneously at six hospitals or clinics in Spain. A paired-match analysis was then performed looking for associations and odds ratios in sociodemographic characteristics, pathological background, clinical presentation and analytical details. Cases outcomes after a six-month follow-up visit were also registered and their particularities described. Most cases and controls came from Latin America (63%–47%) or sub-Saharan Africa (26%–35%). The number of years residing in Spain (9.9 vs. 9.8, *p* = 0.9) and immunosuppression status (30% vs. 36.3%, *p* = 0.2) were also similar in both groups. Clinical symptoms such as diffuse abdominal pain (21% vs. 13%, *p* = 0.02), and epigastralgia (29% vs. 18%, *p* < 0.001); along with a higher eosinophil count (483 vs. 224 cells/mL in cases and controls, *p* < 0.001) and the mean total Immunoglobulin E (IgE) (354 U/L vs. 157.9 U/L; *p* < 0.001) were associated with having strongyloidiasis. Finally, 98.2% percent of the cases were treated with ivermectin in different schedules, and 94.5% met the cure criteria at least six months after their first consultation. Abdominal pain, epigastralgia, eosinophilia, increased levels of IgE and Latin American origin remain the main features associated with *S. stercoralis* infection, although this association is less evident in immunosuppressed patients. The appropriate follow-up time to evaluate treatment response based on serology titers should be extended beyond 6 months if the cure criteria are not achieved.

## 1. Introduction

*Strongyloides stercoralis* is an intestinal nematode that infects an estimated 30–100 million people worldwide [1]. It generally occurs in tropical and subtropical countries, but it might also be present in areas with temperate climate and moist soils [2], hence it has been reported in some areas of Spain [3,4,5]. However, strongyloidiasis is now increasingly emerging due to migration flows from high endemic areas and is a main risk factor for *S. stercoralis* infection [1,6]. There is a public health impact in the non-endemic countries that host these populations. The seroprevalence of *S. stercoralis* can vary substantially depending on the country of origin [7], with the highest incidence from countries such as Cambodia (36%) or Latin American countries (26%) [8]. The few studies conducted in migrants coming from endemic areas showed a prevalence above 9% at hospital level [9], and in studies conducted in non-endemic areas at primary care or community level reported a similar prevalence at around 10% [10,11].

*S. stercoralis* helminth has a particular life cycle resulting in autoinfection in the human host; therefore, the infection can persist for the lifetime of the host if untreated [12]. The infection produces a wide range of clinical symptoms, from asymptomatic ones to more severe clinical syndromes such as hyperinfection or disseminated disease. The latter typically occur in immunosuppressant conditions [13], such as oncological chemotherapy and steroids, and with Human T-lymphotropic virus 1 (HTLV-1) coinfection [14]. However, most infections are asymptomatic, or present with scarce digestive, respiratory or skin manifestations, and therefore strongyloidiasis is often not suspected and underdiagnosed [7].

The risk factors identified for acquiring all forms of strongyloidiasis are HTLV-1 coinfection, malnutrition, chronic obstructive pulmonary disease, diabetes mellitus, chronic renal failure and breastfeeding [14], although such risk factors have not been evaluated in migrant populations.

In our institutions, hospital admissions due to strongyloidiasis have increased tenfold in the last few decades, having a mean cost per patient of EUR 17,122.4 (± 98,000), with an observed crude mortality rate of nearly 8% [15]. In severe cases, the fatality rate can be as high as 63% [16], but can decrease to 11% if the disease is properly managed [17].

The sensitivity of conventional microscopic-based techniques is far from optimal, particularly in chronic infections [12]. Therefore, the diagnosis of strongyloidiasis in non-endemic areas is currently based on a serological test, which has a considerably higher sensitivity compared with standard fecal techniques [12]. Antibody detection in serum is thus the current recommended screening technique to detect the presence of *S. stercoralis* in those coming from an endemic area [18], especially if presenting clinical symptoms or eosinophilia. The negativization of the serology and the decrease by more than half of the baseline optical density of the serological titers are considered cure criteria [18]. The disappearance of clinical symptoms if present and the normalization of the absolute eosinophil count could be considered response-to-treatment indicators, but not cure criteria [19]. 

Identifying patients with high-risk factors for both chronic and severe disease is key to prevent the complications of the disease and reduce mortality. The aim of this study was to determine the factors associated with strongyloidiasis in migrants living in a non-endemic area and to assess determinants for response to treatment in those diagnosed with the infection.

## 2. Results

During the study period, 2024 individuals attending six hospitals were screened for strongyloidiasis. Results of the seroprevalence data published elsewhere [9] showed an overall seroprevalence of *S. stercoralis* of 9.04% (95%CI 7.76–10.31). 

There were 186 patients diagnosed with *Strongyloides stercoralis* infection who met the inclusion criteria for this study. More than half came from Tropical Medicine and International Health services (102, 54.8%), and the remaining ones came from Infectious Diseases departments (58, 31.18%), Pneumology departments (9, 4.8%), Rheumatology or Systemic diseases departments (8, 4.3%), Oncology and Haematology departments (6, 3.2%) and transplant units (3, 1.6%). They all received information about the study and were requested to give consent. Two matched controls by case were then identified from each service/department. In our final sample, 148 (30.6%) patients came from Hospital Clinic, 111 (23%) from Hospital de Poniente, 93 (19.3%) from Hospital Vall de Hebron, 68 (14.1%) from Hospital Sant Pau, 33 (6.8%) from Hospital del Mar and 30 (6.2%) from Hospital de Bellvitge. 

After excluding 11 cases and 30 controls owing to multiple reasons (withdrawn consent for the second study, lack of adequate paired-matching or those with more than 10% of data missing) a sample of 158 cases and 294 controls were included in the final analysis. Additional information on included individuals is provided in Table 1.

The mean age was 39.9 years (SD 12) and 44% were female. Regarding origin, 100 cases and 137 controls (63% and 47%) were from South American countries followed by 41 (26%) cases and 102 (35%) controls from sub-Saharan African countries. A total of 84% of the participants came from a country with a low level of open defecation (see Materials and Methods for open defecation classifications). Out of 158 cases, 29 (18.4%) were immunosuppressed. The main causes of immunosuppression among these 29 cases were as follows: Human immunodeficiency viruses (HIV) infection with a low CD4 lymphocyte count (11 below 500 cells/mm^3^ and 1 below 200 cells/mm^3^; 12, 41.4%), autoimmune disease (8, 27.6%), malignancies (6, 20.7%), receiving oncology chemotherapy (2, 6.9%) and receiving transplants (3, 10.3%).

### 2.1. Crude Analysis 

No differences between cases and controls were observed according to mean age, gender, pregnancy condition, recruitment site (service), number of years residing in Spain, immunosuppression or HIV status (see Table 1 and Table 2). There were differences according to geographic area, with cases more frequently reported from South American countries compared with controls (*p* = 0.002). However, there were no significant differences among cases and controls when the country of origin was considered of high level for open defecation (10% vs. 13%, *p* = 0.6). 

The clinical symptoms that were associated with strongyloidiasis were diffuse abdominal pain (21% vs. 13%, *p* = 0.02), epigastralgia (20% vs. 7%, *p* < 0.001) and urticaria (16% vs. 7%, *p* = 0.005). No other clinical symptoms were associated with having strongyloidiasis (See Table 2). 

**Figure 1:**
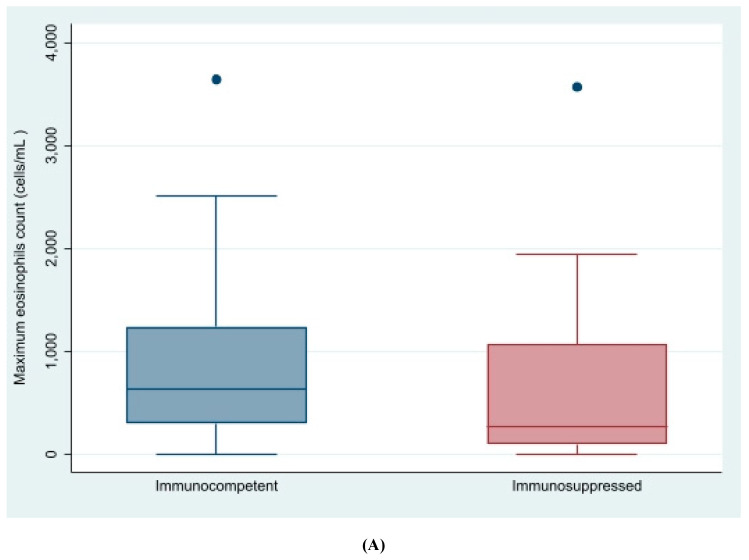

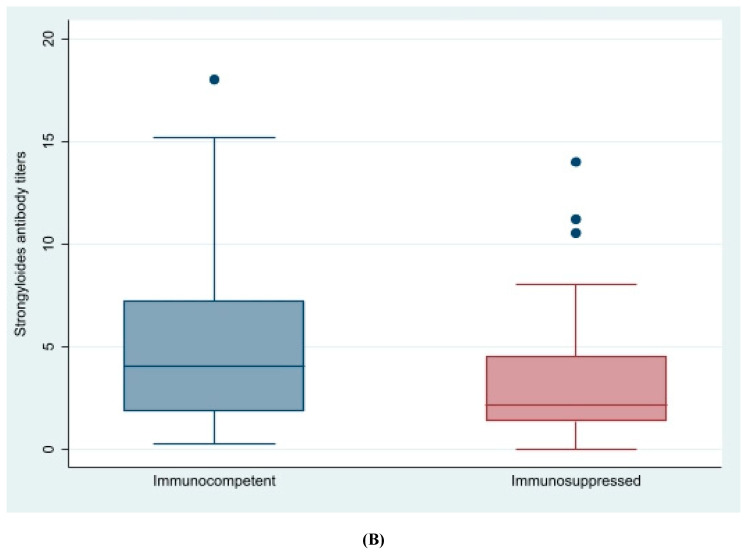
Eosinophil count and *Strongyloides* antibody titers in cases with strongyloidiasis by immunosuppression status. (**A**): Eosinophils count in patients with strongyloidiasis by immunosuppression status. (**B**): Strongyloides antibody titers in patients with strongyloidiasis by immunosuppression status

The mean absolute maximum number of eosinophils was higher in cases compared with controls (494.8 cells/mL vs. 220.5 cells/mL; *p* < 0.001), as was the mean total IgE (354 U/L vs. 157.9 U/L; *p* < 0.001). Similar findings were observed when eosinophilia was considered as a percentage of the total leucocyte count (29.13% vs. 5.5%, *p* = 0.03). Overall, immunosuppressed patients with strongyloidiasis tended to present less eosinophilia (44.8% vs. 62.8%, *p* = 0.07), a lower eosinophil count (median 270 vs. 630, *p* = 0.07) and a lower serology titers than those not immunosuppressed (median 2.1 vs. 4.02 *p* = 0.04, Wilcoxon rank-sum test) (see Figure 1A,B). Co-infections with other parasitic bowel infections was not associated with a diagnosis of strongyloidiasis (10% vs. 11% in cases and controls, respectively; *p* = 0.7). 

### 2.2. Multivariable Analysis

A logistic regression model was built including the sex, age, geographic area, maximum eosinophils, epigastralgia, diffuse abdominal pain, and immunosuppression status of the individual. There was an association between strongyloidiasis and having eosinophilia (adjusted OR 5.6, CI 3.6–8.8, *p* < 0.001), and the geographic area, with coming from Latin American (LA) countries being more frequent (adjusted OR for African countries 0.4, CI95%: 0.3–0.7, *p* = 0.002; adjusted OR for Asian countries 0.2, CI 95%: 0.06–0.09, *p* = 0.03). 

### 2.3. Response to Treatment and Outcome 

Treatment was administered to 158 cases (as prescribed by the treating hospital protocol) and included ivermectin 200 µg/Kg per day, single dose (16, 10.1%), two consecutive days (117, 74.1%), four doses split in two weeks (18, 11.3%), or more than four doses (1, 0.6%), albendazole 400 mg/12 h for seven days (3, 1.9%) and ivermectin plus albendazole (3, 1.9%). 

Among all positive cases, 131 completed a follow-up visit, scheduled around six months after treatment onset although a serological test was performed only in 117 patients, included to evaluate treatment cure. The median time to this second appointment was 209 days (Interquartile range (IQR) 184–248), but uncured patients did the follow-up test before those considered cured (*p* = 0.044). 

At the follow-up visit, 96 (82.1%) patients had achieved negative serology, and a further 15 (12.8%) had decreased their *S. stercoralis* titers by more than half from the baseline level, and therefore fulfilled the cure criteria, which gives an overall 94.9% cure rate. The median *S. stercoralis* titer at this follow-up visit was 0.4 (IQR 0.1–0.7). Six (5.1%) patients did not meet the serological “cure” criteria. There was no association between the negativization of the serology and time elapsed from treatment advice and the follow-up visit (median 210 vs. 232.5 days, *p* = 0.8). Three out of six uncured patients were presenting with co-infections (see Figure S1), although having a co-infection was not associated with being uncured. 

The main characteristics of cured and uncured patients are described in Table 3.

Two of the uncured patients (33.3%) had active immunosuppressive conditions: one was HIV positive with indetectable viral load but had been diagnosed with a Muco-cutaneous Kaposi syndrome one year ago. The other individual had had an autoimmune disease, and was on long-term steroids (there was also one undergoing rituximab treatment). Nevertheless, no difference was observed among cured and uncured patients regarding their immune status.

In addition, 47 patients with strongyloidiasis presented evidence of other parasitic infections at the time of diagnosis, in six cases multiple infections. We found 13 (27.7%) cases of *Trypanosoma cruzi*, 16 schistosomiasis (34%), four of *Echinococcus* spp (8.5%), two of *Fasciola hepatica* (4.2%), three of *Toxocara* (3, 6.4%) and four of *Mansonella perstans* (8.5%). In addition, a fecal test allowed the identification of one case of *Entamoeba histolytica* (2.1%), six of hookworm (12.8%), two of *Giardia duodenalis* (4.3%) and one case of *Taenia* spp (2.1%), *Ascaris* (2.1%) and *Trichuris* (2.1%) respectively. Another six presented non-pathogenic amoebae in feces. All patients without a cure criterion presented an eosinophil count below 450 cells/mL.

## 3. Discussion

In our study, having strongyloidiasis was associated with diffuse abdominal pain, epigastralgia, eosinophilia and increased levels of IgE. These clinical and laboratory findings are consistent with other studies [20] showing that eosinophilia is a good correlator of the infection. Sociodemographic characteristics such as age, sex and country of origin are in accordance with those found in our non-community migrant population [21], except for a slightly higher presence of men, which might reflect the occupational nature of the infection [22]. On the other hand, we also found a weak association with the geographical area of origin being that the infection was more frequently reported in individuals coming from Latin American countries. There was however no association between strongyloidiasis and migrant people coming from countries with a high percentage of open defecation, which could potentially be associated with a higher risk of infection due to higher exposure [23].

The number of years residing in Spain was also not associated with having strongyloidiasis. This may be also explained by the auto-infective *Strongyloides* cycle that can lead to chronic infection if untreated. Therefore, risk should not be perceived to decrease with time, as has already been observed in studies conducted with war veterans, who showed strongyloidiasis several years after leaving the endemic area [24]. In our work, HIV infection and other parasitic infections in stool were also not associated with a higher risk of presenting *Strongyloides stercoralis* infection, but might have a role in decreasing titers after treatment.

Immunosuppression has not been demonstrated to be associated with a higher risk of chronic strongyloidiasis. However, the sensitivity of the serological test for patients receiving immunosuppressant therapy may be lower, although our results were not statistically significant [12]. In our study, after excluding HIV immunocompetent cases, 37 cases were considered immunosuppressed, but due to the study design and logistical reasons, only a serological test was performed and no additional direct parasitological techniques were performed in most of the patients. This may have resulted in missing some *Strongyloides* cases since the serology in these patients is less sensitive [18]. Although no association was found with respect to immunosuppression and response to treatment, it is worth saying that a lower although weakly significant serology titer at baseline was found in the immunosuppressed group. 

Most patients were treated with different ivermectin treatment regimens according to the recommended clinical practice in each hospital [25] and almost all of them achieved the cure criteria. The 5% not cured consisted of patients for whom the follow-up visit was scheduled around a month earlier than for those who were cured, suggesting that a wider follow-up might be needed, in accordance with other findings for migrants coming from endemic areas [26]. It should be added that the follow-up period was up to six months. Although the difference was not significant due to unbalanced numbers, there was a shorter follow-up period (time to visit in days) for the uncured versus the cured patient group. It remains unclear if the follow-up period should be extended to12 months to reach the proposed cure criteria [18], but it could definitely be extended if the cure criteria have not been achieved at six months. As described elsewhere, eosinophilia and IgE will normalize in time after treatment [19].

### Limitations of the Study

One of the main limitations of this study was the lack of a specific gold standard test for the diagnosis of strongyloidiasis, particularly in immunosuppressed patients. Therefore, the absence of association between strongyloidiasis and immunosuppression could be due to a low detection of *Strongyloides* antibodies in immunosuppressed patients. Future studies are needed to report the sensitivity of the serological test under different immunosuppressant conditions. In such cases, the role of Polimerase chain reaction (PCR) tests compared with other direct parasitological techniques such as culture or the Baermann method looks promising, but their performance in immunosuppressed individuals needs further evaluation [27].

In immunosuppressed patients both a serological test and parasitological techniques may increase diagnosis sensitivity [28], although further evidence is needed to establish the most appropriate strategy to detect the infection under immunosuppression conditions. In addition, an HTLV-1 serological test was only performed in 1 out of 269 controls; therefore, it could not be associated with the acquisition of strongyloidiasis and could not be included in the multivariate model.

Another limitation was the retrospective design of the study. On the other hand, the case-control study design was matched by admission unit to minimize the potential bias. Also, occupational and travel risk factors were not obtained for the patients and may have been cofounders.

## 4. Materials and Methods

### 4.1. Study Population, Data Collection and Patient Management

This was a case-control study, carried out as part of a hospital-based prospective cohort study conducted in six referent hospitals in Spain (Hospital Clinic, Hospital del Mar, Hospital Universitari de Bellvitge, Hospital de la Santa Creu i la Sant Pau and Hospital Universitari Vall d’Hebron located in Barcelona and Hospital de Poniente in the Almeria province) from October 2014 to September 2016. Individuals at risk of being infected with *S. stercoralis* were identified (namely those having resided in an endemic country for more than one year, and having attended the hospital for any reason) and a systematic screening for *S. stercoralis* was offered to patients attending any of the six participating hospitals. This screening was performed in the tropical medicine and international health, transplant, haematology and oncology, pneumology, infectious diseases (including HIV) and systemic diseases departments (including rheumatology and dermatology) of each hospital [9]. However, it should be added that although not all patients were immunosuppressed, the reasons for hospitalization in those units were always conditions related to such units. The endemic countries were considered to be any country in Asia, Oceania, Africa, eastern Europe, or Latin America. Therefore, the screening test was offered to those having resided in an endemic country for more than one year and having attended the hospital for any reason. The serological test was a commercial enzyme-linked immunosorbent assay (ELISA), which has been described elsewhere [9]. A normalized optical density (OD) was used, with an OD/cut-off ratio > 1 being reported as positive.

Briefly, patients diagnosed with strongyloidiasis were invited to participate in the study. The inclusion criteria were age above 18, being able to give written consent and being positive for *S. stercoralis* serology and/or parasitological determination. All participants were provided with treatment according to the guidelines or recommendations of each hospital. Participants were clinically and microbiologically followed up with a serological test and with three stool tests (if the initial stool test was positive) six months after the therapy as part of routine clinical practice. The sample size was estimated based on a previous systematic review and meta-analysis of strongyloidiasis prevalence in migrants that reported a random prevalence of 11% [29]. Based on these data, at least 1700 individuals were estimated to be screened over 24 months to recruit 150 cases of strongyloidiasis, considering an alpha error of 5% and a loss to follow-up rate of 10%, as explained in previous work by this group [9].

For the case-control sub-study, all recruited positive patients were considered cases. A sample of 300 screened patients who tested negative following the same screening program were used as controls whenever the above-mentioned test gave a negative result and patients were considered to be migrants coming from endemic areas and fulfilling the inclusion criteria.

Cases and controls were matched by each service and hospital. Every case was matched to two controls (1:2) according to the service that performed the screening and using consecutive selection criteria.

Data were collected through a questionnaire that included age, sex, migration status, country of origin, any symptoms at presentation, and other variables related to immunosuppression. We also collected data on the presence of eosinophilia, results of stool testing, HIV and HTLV-1 serological status, and clinical outcomes six months after the therapy. Eosinophilia was defined as a recorded eosinophil count > 499 × 109 cells/mL.

Countries of origin where grouped into geographical areas according to the Geosentinel database classification [30]. An open defecation variable was also defined as the percentage of a country’s inhabitants who regularly used this sanitation method predominantly, following the criteria of the World Health Organization (WHO) and the United Nations Children’s Fund (UNICEF) [31]. This percentage was categorized into low (0%–33%), medium (34%–66%) or high (67%–99%).

Serological titers were measured at baseline and at least six months after treatment administration. Patients were considered cured when the serological test seroconverted to negative (<1) or when the titers at this follow-up visit had decreased by at least 50% compared with the baseline test.

### 4.2. Data Analysis

Descriptive analysis was performed in terms of arithmetic mean, standard deviation (SD), median, inter-quartile range and percentage as appropriate. Given the 1:2 matched case-control design, each matched case was paired with the corresponding two controls in order to calculate differences between the cases and controls. Those differences were described in terms of mean and standard deviation (SD) for continuous variables, and number and proportion of discrepant pairs for categorical variables. Crude associations between cases and controls were calculated using a paired Student’s *t*-test, an exact McNemar’s test or a symmetry (asymptotic) test as appropriate.

Conditional multivariate logistic regression was fitted using a conditional logistic regression analysis with backward stepwise procedures to investigate the association between strongyloidiasis and immunosuppression adjusted for statistically significant variables from the univariate analysis. This was performed based on the Wald test estimates with a 0.05 significance level for removal from the model. Those variables that had more than 10% missing values were not considered suitable candidates for the multivariate analysis. The multivariate analysis was performed using the complete records for the preselected variables (complete case analysis) assuming missing completely at random (MCAR). A second multivariate model was estimated by forcing the variables identified in the first model and selecting their two-way significant interactions selected by the backward procedure explained above.

In order to evaluate the potential bias introduced by this sample selection, and to assess the need to force some of these variables to be part of the final model, we compared the within-pairs discrepancies between the complete and restricted samples using the Student’s *t*-test or the chi-square test as appropriate.

Statistical significance was established at *p*-value < 0.05 and confidence intervals at 95% level (95%CI). All analyses, data manipulation, and implementations were performed using STATA version 15 (StataCorp, College Station, TX, USA).

### 4.3. Ethical Aspects

The study was approved by every ethical committee from all the hospitals participating in the study (HCB/2014/0321).

## 5. Conclusions

We conclude that abdominal pain, epigastralgia, eosinophilia, increased IgE levels and having Latin American origin are the main factors associated with *S. stercoralis* infection. However, laboratory findings may be less informative when immunosuppression is present. In addition, further studies should elucidate the appropriate time to follow up on treatment response based on the serology titers or to extend it if negativization has not been achieved at 6 months.

## Figures and Tables

**Table 1:** Demographic characteristics of the patients.

Variable Name	Cases *n* = 158	Controls *n* = 294	Discrepancies within Matched Pairs	Overall *p*-Value
Age *	39.88 (SD 12)	44.6 (SD 60.5)	−4.8 (61.4)	0.18
Female gender	70 (44%)	124 (42%)	61/129 (47%)	1.00
Pregnancy	2 (3%)	8 (6%)	1/2 (50%)	0.18
Geographic area:				0.001
South America	100 (63%)	137 (47%)	71/181 (39%) 5	
Central America	4 (3%)	12(4%)	5/8 (62%) 5	
Caribbean	4 (3%)	9 (3%)	7/8 (88%)	
Sub-Saharan Africa	41 (26%)	102 (35%)	18/81 (22%)	
North Africa	4 (3%)	15 (5%)	2/8 (25%)	
South-Central Asia	0 (1%)	12 (4%)	0/0 (%)	
North-East Asia	3 (2%)	4 (1%)	5/5 (100%)	
South East Asia	0 (0%)	0 (0%)	0/0 (0%)	
Europe	2 (1%)	2 (1%)	3/3 (100%)	
Unknown	0 (0%)	1 (0%)	0/0 (%)	
**Open Defecation**				
Low	132 (84%)	236 (80%)	24/230 (10%)	0.45
Medium	16 (10%)	39 (13%)	19/30 (63%)	
Unknown	10 (6%)	19 (6%)	15/15 (100%)	
Years in Spain *	10 (6.2)	9.8 (7.4)	−0.03 (9.6)	0.9

* Quantitative variables expressed as mean and standard deviation.

**Table 2:** Co-morbidities and clinical manifestations of strongyloidiasis.

Variable Name	Cases *n* = 158	Controls n = 293	Odds Ratio (95% CI)	*p*-Value
**Pathological Background**				
Immunosuppressed patient	29/158 (18.4%)	67/293 (22.9%)	0.8 (0.5, 1.2)	0.26
HIV	28/139 (20%)	63/235 (27%)	0.7 (0.2, 2.2)	0.82
>500 CD4	16 (10%)	37 (12.6%)	-	-
500–200 CD4	11 (7%)	21 (7%)	1.1 (0.3, 3.4)	0.93
<200 CD4	1 (1%)	5 (2%)	0.53 (0.05, 5.9)	0.60
Last viral load (copies/mL)	1606 (6352.8)	8302.1 (28174)	*	
HTLV-1 positive serology	2/68 (3%)	0/1 (0%)	*	-
Transplanted	3/158 (1.9%)	7/294 (2.3%)	*	-
Neoplasia	6/137 (4%)	8/206 (4%)	1.4 (0.4, 5.1)	0.6
Other pathogenic parasites in stool	14/144 (10%)	15/200 (8%)	1.76 (0.76, 4.1)	0.19
**Clinical Manifestations and Blood Test Results**				
Diffuse abdominal pain	33/157 (21%)	39/291 (13%)	1.90 (1.1, 3.2)	0.02
Epigastralgia	29/144 (20%)	18/269 (7%)	3.6 (1.8, 7.1)	<0.001
Urticaria/Pruritus	25/158 (16%)	21/291 (7%)	2.5 (1.3, 4.6)	0.005
Maximum eosinophil count cells/µL	494.8 (598.1)	220.5 (213.6)	1.58 (1.4, 1.8)	<0.001
	(*n* = 156)	(*n* = 276)		
Maximum leukocyte count cells/µL (n = 153)	6942.3 (2542.3)	6495.4 (2591.1)	1.1 (1, 1.2)	0.1
	(*n* = 157)	(*n* = 287)		
Total IgE (IU/L) (n = 153)	354 (593.1)	157.9 (237.6)	1.5 (1.2, 1.8)	<0.001
	(*n* = 122)	(*n* = 106)		
HTLV-1 serology	2/57 (4%)	-	-	-

* Please note no estimates are available if total count is below 5.

**Table 3:** Main characteristics of 138 patients who completed the follow-up, according to cure criteria.

Main Variables *n* = 117	Cured = 111	Uncured *n* = 6	*p*-Value
Female gender	48 (43.2%)	3 (50%)	0.7
Age	38.3 (SD 13.1)	33.9 (SD 10.5)	0.2
Geographic area *n* = 117	*n* = 111	*n* = 6	0.06
South America	71 (64%)	2 (33.3%)	
Central America and Caribbean	6 (5.4%)	1 (16.7%)	
North Africa	4 (3.6%)	0	
Sub-Saharan Africa	28 (25.3%)	2 (33.3%)	
South-East Asia	1(0.9%)	1 (16.7%)	
Eastern Europe	1 (0.9%)	0	
Immunosuppressed	37 (30.6%)	2 (28.6%)	0.91
Baseline serology titers	4.3 (SD 3.9)	5. (SD 3.1)	0.15
Other parasitic infections	34 (29.1%)	2 (28.6%)	0.98
Treatment provided included ivermectin (*n* = 124)	117 (98.3%)	7 (100%)	0.64
Time to visit in days	210 (SD 82.9)	232.5 (SD 107.1)	0.8

## Supplementary Materials

The following are available online at https://www.mdpi.com/2076-0817/9/6/507/s1, Figure S1: Co-infections in patients with strongyloidiasis by cure rate.
