# Supplementary figures and images for: Clinical Features Associated with Strongyloidiasis in Migrants and the Potential Impact of Immunosuppression: A Case Control Study
Source: Pathogens. 2020 Jun 23;9(6):507. doi: 10.3390/pathogens9060507 (PMC7350355; doi:10.3390/pathogens9060507)

## SUPPLEMENTARY FILES

Figure S1. Co-infections in patients with strongyloidiasis by cure rate

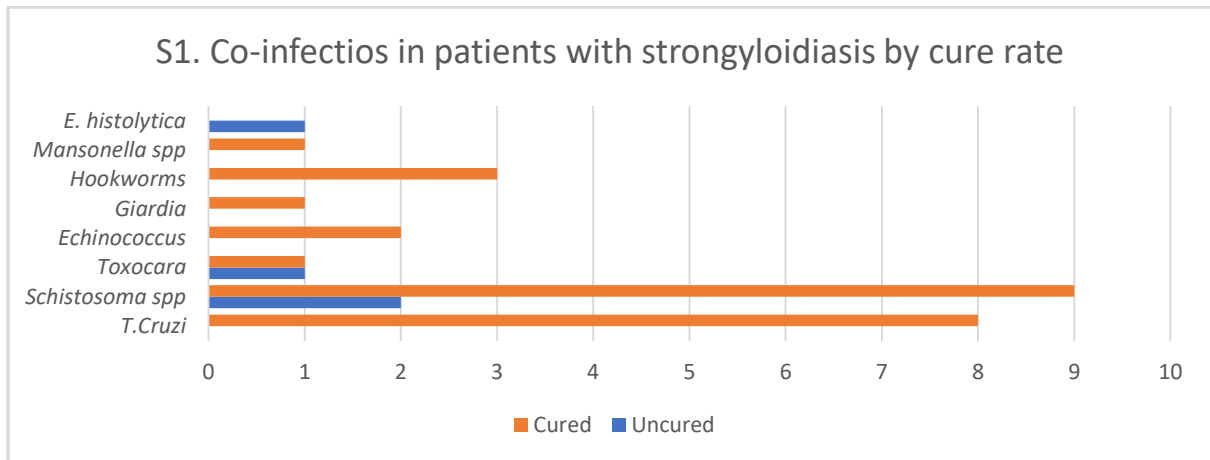

Supplement: Supplementary file 1 [file pathogens-09-00507-s001.pdf]
